# Microwave ablation combined with vertebral augmentation under real-time temperature monitoring for the treatment of painful spinal osteogenic metastases

**DOI:** 10.1186/s12883-023-03263-x

**Published:** 2023-06-08

**Authors:** Jing Fan, Xusheng Zhang, Peishun Li, Linlin Wu, Qianqian Yuan, Yunling Bai, Sen Yang, Yuanyuan Qiu, Kaixian Zhang

**Affiliations:** Department of Oncology, Tengzhou Central People’s Hospital, Affiliated to Jining Medical College, Tengzhou, China

**Keywords:** Osteoblastic metastasis, Refractory pain, Microwave ablation, Vertebral augmentation, Real-time temperature monitoring

## Abstract

**Objective:**

To evaluate the safety and efficacy of computed tomography (CT)-guided microwave ablation combined with vertebral augmentation under real-time temperature monitoring in the treatment of painful osteogenic spinal metastases.

**Methods:**

This retrospective study included 38 patients with 63 osteogenic metastatic spinal lesions treated using CT-guided microwave ablation and vertebral augmentation under real-time temperature monitoring. Visual analog scale scores, daily morphine consumption, and Oswestry Disability Index scores were used to evaluate efficacy of the treatment.

**Results:**

Microwave ablation combined with vertebral augmentation reduced the mean visual analog scale scores from 6.40 ± 1.90 preoperatively to 3.32 ± 0.96 at 24 h, 2.24 ± 0.91 at 1 week, 1.92 ± 1.32 at 4 weeks, 1.79 ± 1.45 at 12 weeks, and 1.39 ± 1.12 at 24 weeks postoperatively (all p < 0.001). The mean preoperative daily morphine consumption was 108.95 ± 56.41 mg, which decreased to 50.13 ± 25.46 mg at 24 h, 31.18 ± 18.58 mg at 1 week, 22.50 ± 16.63 mg at 4 weeks, 21.71 ± 17.68 mg at 12 weeks, and 17.27 ± 16.82 mg at 24 weeks postoperatively (all p < 0.001). During the follow-up period, the Oswestry Disability Index scores significantly reduced (p < 0.001). Bone cement leakage occurred in 25 vertebral bodies, with an incidence of 39.7% (25/63).

**Conclusions:**

The results indicate that microwave ablation combined with vertebral augmentation under real-time temperature monitoring is a feasible, effective, and safe treatment for painful osteoblast spinal metastases.

## Introduction

The incidence of bone metastasis is approximately 20–80% in patients with malignant tumors in the advanced stages [1, 2]. The spine is the most common site of bone metastasis because of its highly vascularized anatomy, with an incidence of approximately 60–70% in patients with advanced cancer [3]. Spinal metastases are observed in approximately 90–95% of patients with severe pain; it can cause pathological fractures, spinal cord compression, or life-threatening hypercalcemia and lead to poor quality of life [4–6]. Treatments for patients with spinal metastases aim to relieve pain, preserve nerve function, and reduce pain-related disability, all of which are usually achieved through minimally invasive treatments [4, 7]. Based on computed tomography (CT) findings of the lesions, 78.3% of vertebral lesions are classified as osteolytic, 20.1% as mixed, and 1.6% as osteogenic [8]. Most of the previous reports on minimally invasive surgery for spinal metastasis have focused on osteolytic metastasis. Previous studies have explored the use of microwave ablation (MWA) combined with bone augmentation for the treatment of osteolytic spinal metastases [5, 6, 9]. This retrospective study aimed to evaluate the efficacy and safety of MWA combined with vertebral augmentation (VA) in the treatment of painful osteoblastic spinal metastases.

## Materials and methods

This retrospective study was approved by the institutional ethics committee. All patients and their families provided informed consent.

### Patients

This study included 38 patients (18 men, 20 women; average age, 60.97 ± 14.15; range, 22–85 years) with osteogenic spinal metastases who had undergone percutaneous MWA and VA from April 2015 to March 2021. The baseline data of the patients are shown in Table 1. All patients underwent CT and magnetic resonance imaging (MRI) before treatment for assessment of the location, size, and nature of the lesions.

**Table 1:** Baseline Characteristics of the Study Participants

Characteristics		Number	Percent(%)
Mean age (years)		60.98 + 14.15(range:22–85)	
SEX			
	Male	18	47.4
	Female	20	52.6
Pathology			
	Lung	14	36.8
	Breast	7	18.4
	Esophagus	5	13.2
	Stomach	4	10.5
	Unknown	3	7.9
	Prostate	2	5.3
	Colon	1	2.6
	Pancreas	1	2.6
	Mediastinum	1	2.6
Pathological type			
	Adenocarcinoma	26	68.4
	Squamous cell carcinoma	9	23.7
	Small cell Carcinoma	1	2.6
	Fibrosarcoma	1	2.6
	Non-Hodgkin’s Lymphoma	1	2.6
Location			
	Thoracic	31	49.2
	Lumbar	32	50.8

### Inclusion and exclusion criteria

Patients with the following characteristics were included: (1) clear pathological diagnosis of primary tumor or vertebral metastasis; (2) focal pain localized to a certain part of the vertebral body (visual analog scale [VAS] scores ≥ 4); (3) osteogenic metastasis type of vertebral metastasis; (4) ≤ 4 lesions under treatment per patient; (5) intractable pain with limited effects of radiotherapy, chemotherapy, and opioid analgesic drugs; and (6) estimated survival time ≥ 12 weeks.

Patients with the following characteristics were excluded: (1) uncorrectable coagulation disorders (platelet count > 50 × 10^9^/L; international normalized ratio > 1.50); (2) uncontrolled local (lesional) or systemic infection; (3) compression of the spine by the tumor and exhibition of symptoms of spinal cord compression; and (4) Eastern Cooperative Oncology Group Performance Status score ˃3.

### Procedure

The patient was guided to the appropriate position on the CT (SOMATOM Definition AS; Siemens Healthineers, Erlangen, Germany) bed. A vacuum-negative pressure pad was used to stabilize the patient’s position, and ECG monitoring was initiated. The positioning grating was placed on the patient’s back, and CT and three-dimensional reconstruction of the spine was performed. The puncture site and method were decided by analyzing the CT scans. General anesthesia with sufentanil (50 µg /mL diluted in a ratio of 1:10 with saline solution) and local anesthesia (1% lidocaine hydrochloride and 0.25% ropivacaine hydrochloride) were combined before surgery.

The optimal site and angle for needle puncture were selected based on the analysis of the CT scan, and a bone drill was used to assist a 13-G bone puncture needle (Shandong Guanlong Medical Instrument Co., LTD., Shandong, China) penetrate the anterior middle third of the vertebral body. The bone needles were inserted unilaterally in 12 vertebral bodies and bilaterally in 51. A 20-G (15 cm puncture) needle was used to inject lidocaine into each layer. Under CT guidance, a bone puncture needle was gradually advanced to the front of the long axis of the affected vertebral body. As the osteoblastic metastatic bone is hard, usually a bone drill or surgical hammer is used to assist the needle insertion. The needle core was withdrawn, and the microwave antenna (14- or 16-gauge, 20 cm in length; ECO Microwave Electronic Institute) was inserted. The bone puncture needle was retracted so that the tip of the microwave antenna was 1.5–2.0 cm beyond the bone puncture needle. A 20-G thermocouple needle was inserted at the ipsilateral foramina for real-time temperature monitoring during ablation. If the temperature increased to 42 °C, the ablation was terminated. The appropriate ablation power and time were selected according to the location, size, and adjacent tissues. In this study, the average ablation power was 30.3 ± 8.2 (range, 20–50) W and ablation time was 3.03 ± 1.16 (range, 1–6) min. During the operation, the patient was constantly asked about the pain and feeling of the lower limbs; the operation was stopped if any special problems arose.

**Fig. 1:**
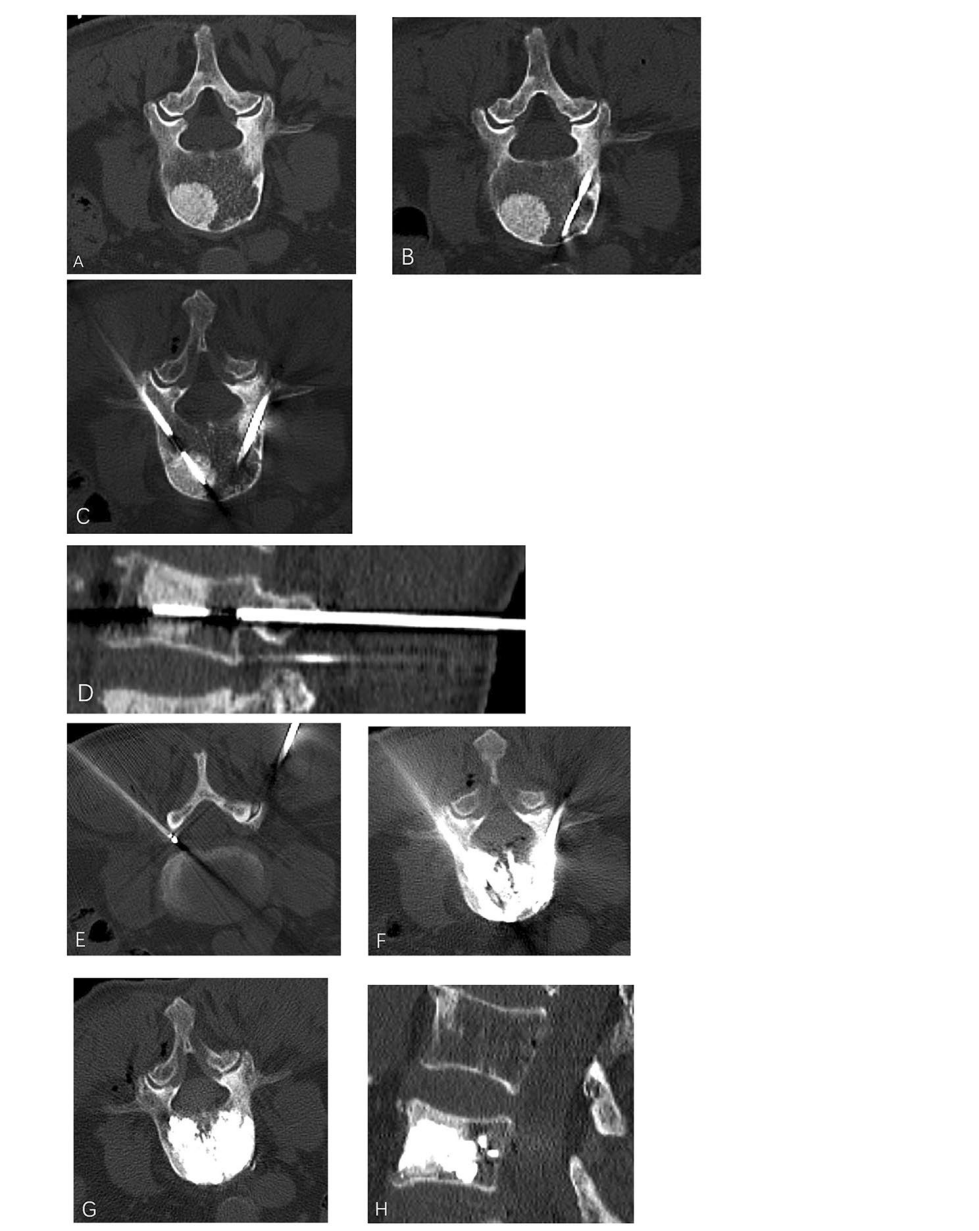
A 72-year-old man with painful osteogenic L4 metastases from prostate cancer was treated with MWA combined with VA. **A**, preoperative axial CT shows L4 osteogenic destruction. **B**-**C**, microwave antennas were placed bilaterally through the pedicle. **D**, an sagittal CT reconstruction after the insertion of the microwave antenna confirmed the position. **E**, a thermocouple was placed in the foramina to monitor temperature changes during MWA. **F**, bone cement was injected bilaterally. **G**-**H**, axial and sagittal CT reconstruction showed bone cement deposits in the lesion area

After ablation, the ablation antenna was withdrawn and the bone puncture needle pushed forward to the appropriate position. After 10 min of ablation, bone cement was injected. We used polymethyl methacrylate (PMMA) cement (Haleus Medical Co., Ltd., Welem, Germany), which was extracted using 1 ml syringes that were placed in ice water to extend the setting time of the PMMA. To reduce the risk of external infiltration, the PMMA bone cement (up to 1 ml) was injected into the lesion slowly, intermittently, and incrementally after ablation. After each injection, CT examinations were performed to observe the degree of filling and flow direction of the bone cement. The amount of the bone cement did not exceed 0.3 ml for the level to be close to the posterior edge of the vertebral body. Once cement leakage into the spinal canal and foramina was detected, the injection was stopped immediately. The amount of bone cement injected depended on the size and location of the tumor. After all surgeries, extensive CT scans were performed to check for complete filling or bone cement extravasation (Fig. 1). The average amount of bone cement injected in each vertebra was 4.98 ± 2.17 (range, 2–8) ml.

### Follow-up

Due to the relatively short duration of survival after spinal metastases, our follow-up duration was 24 weeks. The severity of pain experienced was quantified (expressed in VAS scores, 10: strongest pain, 0: no pain). Oswestry Disability Index (ODI) scores and daily consumption of morphine equivalent before and 24 h, and 1, 4, 12, and 24 weeks after MWA combined with VA were recorded. The patients completed a self-report questionnaire, which assessed the VAS scores, daily consumption of analgesics, and ODI scores. CT and MRI scans of the vertebral body were performed four weeks after surgery and then every 12 weeks to evaluate local progression.

### Statistical analysis

Statistical analysis was performed using SPSS version 23.0 (IBM Corp., Armonk, NY, USA). Continuous variables are expressed as mean ± standard deviation. VAS, ODI scores, and daily morphine consumption were analyzed by paired Student’s t-test. P < 0.05 indicates statistical significance.

## Results

Technical success was defined as placing the MWA antenna at the predetermined position, achieving ablation time and power, and injecting sufficient bone cement into the lesion with satisfactory distribution [5, 6]. This study achieved technical success (100%) in all patients with 63 metastatic vertebrae.

### Pain relief

**Fig. 2:**
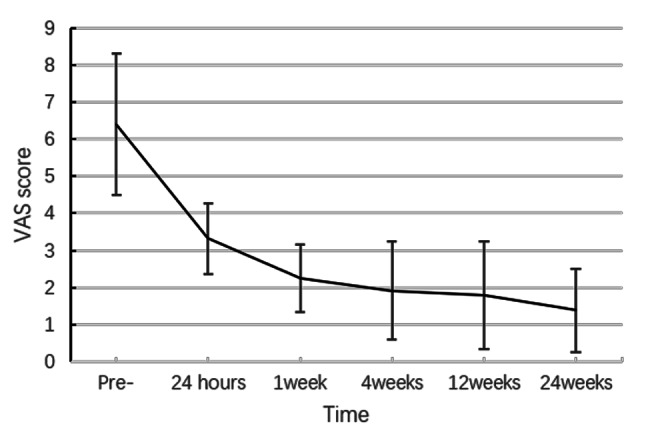
VAS scores before (pre) and after the procedure. VAS, visual analog scale

The pain in all patients was significantly relieved in time or continuously. The mean baseline VAS scores (6.40 ± 1.90; range, 4–10) were significantly greater than the mean VAS scores at 24 h (3.32 ± 0.96; p < 0.001), 1 (2.24 ± 0.91; p < 0.001), 4 (1.92 ± 1.32; p < 0.01), 12 (1.79 ± 1.45; p < 0.01), and 24 weeks (1.39 ± 1.12; p < 0.01) postoperatively (Fig. 2; Table 2).

**Table 2:** Visual Analogue Scale Scores, Daily Morphine. Equivalent Requirement, Oswestry Disability Index Scores

		Value, mean ± SD	P Value
Visual analogue scale score			
	Preoperative	6.40 ± 1.90	<0.0001
	24 h	3.32 ± 0.96	<0.0001
	1week	2.24 ± 0.91	<0.0001
	4weeks	1.92 ± 1.32	<0.0001
	12weeks	1.79 ± 1.45	<0.0001
	24weeks	1.39 ± 1.12	<0.0001
Daily morphine equivalent			
requirement (mg)			
	Preoperative	108.95 ± 56.41	<0.0001
	24 h	50.13 ± 25.46	<0.0001
	1week	31.18 ± 18.58	<0.0001
	4weeks	22.50 ± 16.63	<0.0001
	12weeks	21.71 ± 17.68	<0.0001
	24weeks	17.27 ± 16.82	<0.0001
Oswestry disability index			
score			
	Preoperative	39.71 ± 4.95	<0.0001
	4weeks	24.45 ± 4.17	<0.0001
	12weeks	19.71 ± 3.33	<0.0001
	24weeks	17.46 ± 3.48	<0.0001

**Fig. 3:**
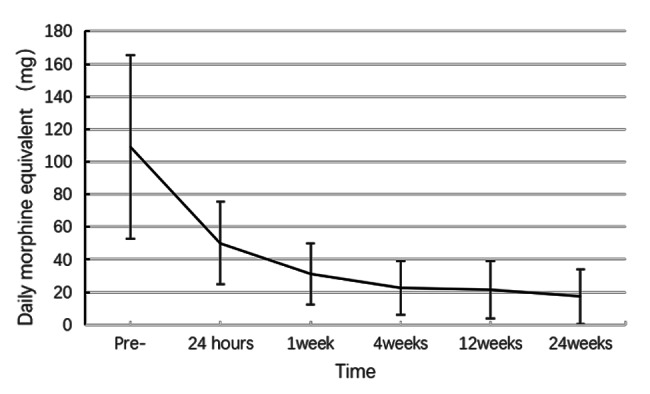
Daily morphine consumption before (pre) and after the procedure

The daily dosage of morphine required was 108.95 ± 56.41 (range, 30–210) mg before the operation, which was significantly higher than that required postoperatively after 24 h (50.13 ± 25.46 mg; p < 0.001), 1 (31.18 ± 18.58 mg; p < 0.001), 4 (22.50 ± 16.63 mg; p < 0.001), 12 (21.71 ± 17.68 mg; p < 0.001), and 24 weeks (17.27 ± 16.82 mg; p < 0.001) (Fig. 3; Table 2).

### Degree of disability

**Fig. 4:**
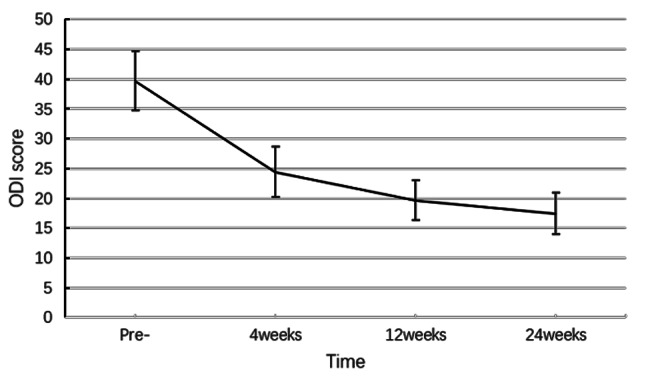
ODI scores before (pre) and after the procedure. ODI, Oswestry Disability Index

The preoperative mean ODI score was 39.71 ± 4.95, which was significantly higher than the scores at 4 (24.45 ± 4.17; p < 0.01), 12 (19.71 ± 3.33; p < 0.01), and 24 weeks (17.46 ± 3.48; p < 0.01) after the procedure (Fig. 4; Table 2).

### Complications

Complications at each patient’s postoperative follow-up time point and the severity of adverse events due to antitumor therapy were assessed using the Common Terminology Criteria for Adverse Events (CTCAE-version 5.0). Bone cement leakage occurred in 25 vertebral bodies (CTCAE grade 1), with an incidence of 39.7% (25/63); of these, no patients showed any obvious symptoms of nerve or spinal cord compression.

## Discussion

The different cytokines released by tumor cells in the bone-tissue microenvironment can lead to the differences between osteogenic and osteolytic metastases [10]. Osteolytic bone metastasis is caused by the release of osteoclast factors from tumor cells in the bone microenvironment [11, 12]. Osteogenic metastasis is caused by the uncontrolled release of bone morphogenetic factors after the proliferation and differentiation of osteoblasts stimulated by metastatic cancer cells [13–15]. Platelet-derived growth factor, urokinase, Endothelin-1, and prostate specific antigen have been reported to be involved in the process of osteoblast metastasis [3]. Osteogenic metastases may increase bone mineral density, but disorders of bone growth may lead to pathological fractures [10]. Moreover, osteogenic metastases can also cause severe pain, spinal paraplegia, and other complications that seriously affect the patient’s quality of life [10]. Therefore, the primary goals of treatment for osteogenic spinal metastases are pain control and prevention of spinal nerve compression and pathological fractures to improve the patient’s quality of life.

Vertebroplasty is an established treatment for spinal metastases. Galibert et al. reported its use in clinical practice in 1987, and it has been widely used in patients with painful vertebral metastases since [16]. Asymmetry in vertebral compression can lead to shear stress fractures and cause pain, and this asymmetry can be corrected by injecting bone cement on the nonosteogenic side [17]. However, PMMA and the exothermic reaction generated during its polymerization cannot completely destroy the tumor cells [18]. Furthermore, an increase in intrametastatic pressure caused by the cement injected in a vertebral body with metastasis accompanied by a risk of dissemination of tumor cells have been reported [19]. The most common complication of bone augmentation is cement leakage, with an incidence of approximately 61.17% [20]. Most cement leaks, such as intervertebral disc and paravertebral leaks, are asymptomatic. However, some intraspinal leaks can lead to severe nerve damage. If cement leaks into the blood vessels, it can lead to pulmonary embolism or perforation of the heart [20].

Calmels et al. performed PVP in 52 patients with 53 osteoblastic metastases and 50 mixed spinal metastases; the rate of pain relief at 1 and 6 months after surgery was 86% and 92%, respectively, while the rate of cement leakage was 50.5% (52/103) [21]. Microwave ablation uses the microwave magnetic field released by the MWA needle to rotate the polar molecules and charged particles around at high speed, which causes tissue solidification, dehydration, and necrosis through friction heating [22]. Theoretically, MWA can achieve better tissue penetration than other types of thermal energy in the treatment of bone metastases owing to the low electrical conductivity of bone tissue [23]. Compared with radiofrequency ablation, MWA has the characteristics of high ablation frequency, strong penetration, and multi-needle joint ablation; therefore, MWA has faster thermogenesis, higher temperature in the tumor, shorter ablation time, and larger ablation area [22]. Radiofrequency ablation is reportedly not suitable for osteogenic metastases because the electrode needle is difficult to open in osteogenic lesions [24]. Cazzato reported that the incidence of transient pain after MWA was approximately 4%, much lower than the 30% rate of radiofrequency ablation [25]. Although ablation alone can destroy the tumor and shrink the tumor tissue, the residual cavity after ablation can cause vertebral compression fracture and spinal stenosis, and the risk of pathological fracture after ablation is about 15–40% [23, 26]. Therefore, some scholars have used MWA combined with bone augmentation in the treatment of spinal metastases [2, 5, 6, 9].

The main advantage of MWA combined with bone augmentation is the ideal distribution of bone cement, especially in invasive tumors that invade surrounding tissues. Optimal cement distribution can reach the affected part of the bone and enhance the efficacy of MWA [6]. At present, the clinical evidence of MWA combined with bone augmentation in the treatment of spinal osteoblastic metastases is limited to a few studies. In this study, VAS scores and daily morphine consumption were significantly less at 24 h postoperatively than those preoperatively. The data were significantly different between the two groups before and after the operation (p < 0.0001), which was maintained until the 24-week follow-up. The ODI scores were significantly lower at 4 weeks postoperatively and remained relatively low during the follow-up period. The results of this study are consistent with those of previous studies [27, 28]. Because the thickening of the trabecular bone in the vertebral body increases the hardness of the bone, it is difficult to use the bone puncture needle alone. Thus, in this study, a bone drill or surgical hammer to aid the entry of the bone puncture needle into the vertebral body was used.

The main complication of single vertebroplasty for spinal metastases is bone cement leakage, the incidence of which can be as high as 50–80% in patients with spinal metastases and compression fractures [29, 30]. The occurrence of cement extravasation is not related to the type of lesion, and in osteoblastic metastatic lesions, it occurs suddenly and cannot be predicted at the beginning of cement injection [21].

In this study, the bone cement leakage rate was significantly lower than that after single vertebroplasty, which was 39.7% (25/63). MWA with VA is associated with a significantly reduced bone cement leakage rate. The proposed mechanisms are tumor shrinkage after ablation leading to the formation of a thermal cavity, which reduces the volume of material in the ablation zone and creates space for the distribution of the bone cement [28]. Additionally, thrombosis may be caused by the ablation, reducing the leakage of bone cement into the vertebral vein and posterior wall [27].

The most serious complication of MWA with VA for spinal metastases is spinal nerve injury, especially in cases with marginal metastatic tumors of the vertebral body. As the ablation zones are larger due to undefined peripheral boundaries, the overheated environment around the ablation area, caused by the increase in the internal temperature by the microwaves, may cause nerve damage [31]. Accordingly, spinal neuroprotective measures should be used for the treatment of spinal metastases via MWA. Thermal protection methods include carbon dioxide or physiological saline isolation and temperature and electrophysiological monitoring [32]. In the present study, real-time temperature was monitored in some patients during the MWA procedure. The safe temperature range for nerves is 15–42 °C [33]; during the ablation process, we ensured our electrodes were below 42 °C. When the monitored temperature exceeded the upper safe limit, the operation was stopped immediately. The highest instantaneous temperature measurements at the level of the neural foramen was 40.5 ± 0.8℃. No cases of spinal cord nerve injury occurred in our study, as evident through postoperative observation and follow-up.

## Limitations

First, a control group was not created and the monitoring data between single vertebroplasty and MWA with VA were not compared. Second, microwave antennas are straight and cannot be bent like RF electrodes. Therefore, adequate ablation of lesions posterior to the center of the vertebral body is difficult. Third, the sample size was small, and further research on this technology should be performed with a larger sample size.

In conclusion, CT-guided MWA combined with VA under real-time temperature monitoring is a feasible, effective, and safe treatment method for osteogenic spinal metastases. It can provide rapid and constant pain relief, control the growth of local tumors, and improve the quality of life of patients.

## Data Availability

The datasets used and/or analysed during the current study are available from. the corresponding author on reasonable request.
